# Hydroxyapatite Nanoparticles in Drug Delivery: Physicochemistry and Applications

**DOI:** 10.3390/pharmaceutics13101642

**Published:** 2021-10-09

**Authors:** Sofía Lara-Ochoa, Wendy Ortega-Lara, Carlos Enrique Guerrero-Beltrán

**Affiliations:** 1 Tecnologico de Monterrey, Escuela de Ingeniería y Ciencias, Monterrey 64710, Mexico; sofialaraaa98@gmail.com; 2 Tecnologico de Monterrey, Escuela de Medicina y Ciencias de la Salud, Medicina Cardiovascular y Metabolómica, Monterrey 64710, Mexico

**Keywords:** hydroxyapatite nanoparticles, physicochemical properties, drug delivery, characterization, biological systems, biomedicine

## Abstract

Hydroxyapatite (HAP) has been the gold standard in the biomedical field due to its composition and similarity to human bone. Properties such as shape, size, morphology, and ionic substitution can be tailored through the use of different synthesis techniques and compounds. Regardless of the ability to determine its physicochemical properties, a conclusion for the correlation with the biological response it is yet to be found. Hence, a special focus on the most desirable properties for an appropriate biological response needs to be addressed. This review provides an overview of the fundamental properties of hydroxyapatite nanoparticles and the characterization of physicochemical properties involved in their biological response and role as a drug delivery system. A summary of the main chemical properties and applications of hydroxyapatite, the advantages of using nanoparticles, and the influence of shape, size, functional group, morphology, and crystalline phase in the biological response is presented. A special emphasis was placed on the analysis of chemical and physical interactions of the nanoparticles and the cargo, which was explained through the use of spectroscopic and physical techniques such as FTIR, Raman, XRD, SEM, DLS, and BET. We discuss the properties tailored for hydroxyapatite nanoparticles for a specific biomolecule based on the compilation of studies performed on proteins, peptides, drugs, and genetic material.

## 1. Introduction

A biomaterial is a synthetic material used to replace part of a living system or a material meant to be in contact with living tissue [1,2]. In this sense, biomaterials can be categorized into polymers, liposomes, micelles, dendrimers, and calcium phosphate (CaP) nanoparticles, where each will show a different type of bioactivity [3]. Bioceramics are known for their high biocompatibility, high chemical stability, and high mechanical strength in vivo [4,5]. Hydroxyapatite (HAP), chemically known as Ca_10_(PO_4_)_6_(OH)_2_, is a biocompatible, osteoconductive, and bioactive ceramic with the ability to form direct bonds with living tissues [6,7]. Although the use of these inorganic nanoparticles poses controversy regarding its safety, the biodegradability and biocompatibility of HAP are significantly better than other nanoparticles [8]. Compared with polymers, HAP poses a pH-dependent dissolution and a higher biocompatibility [9]. HAP also has higher stability than liposomes and micelles, which tend to dissipate under specific concentrations [10]. Moreover, HAP is soluble and less toxic than SiO_2_, TiO_2_, quantum dots, carbon nanotubes, and other magnetic particles [10,11,12,13]. HAP has been the subject of study over the years in the field of biomaterials science due to its importance in clinical applications and tissue engineering [14]. As HAP is the most stable CaP under physiological conditions, it is considered a suitable carrier for the controlled release of compounds. [15,16] In bones and teeth, HAP provides rigidity and corresponds to the main component [17]. Due to its close chemical and physical resemblance to mineral enamel, HAP plays an excellent role in biomedical applications. It has been used to develop artificial bone and teeth and to make biomaterials based on nanoparticles and nanocomposites. HAP also has a low solubility in physiological pH and is used as a carrier for the local delivery of drugs through surgical placement and injection. For this purpose, three critical forms of HAP can be mainly used [18]:

(1) Drugs conjugated or loaded with implanted HAP scaffolds;
(2) Porous HAP or nano-HAP granular particles;
(3) Polymer-coated HAP or nano-HAP particles.

Nanotechnology introduced the concept of nanostructured bioceramics, for which HAP nanoparticles (with at least one dimension of 100 nm) have shown excellent biocompatibility and biodegradability [19]. The surface of the nanoparticles can be altered to allow hydrophobic interactions, charge density, and pi-electron density. Additionally, because of their large surface-to-volume ratios, the nanoparticle’s design significantly influences the interactions with the biological environment [20]. As a biomaterial in the bone system, HAP exerts a better biocompatibility due to its similarity with the bone mineral structure, consisting of tiny HAP crystals at the nanoscale. This characteristic has been shown to be more efficient in osteoblast adhesion and proliferation [6]. Although HAP microparticles have been studied for the release of proteins, since the sizes of the microparticles are not much smaller than those of cells, they are not able to penetrate the cell membrane and are degraded by phagocytosis [4,16].

The use of nano-HAP has been recently justified because, reported as the primary mineral phase of bone, HAP is found as a nanocrystal within a collagen triple helix structure [21]. Pure HAP nanoparticles and HAP nanoparticles combined with polymers have been used for drug delivery applications, and the association of their physicochemical properties with biological applications has become an area of immense research interest. However, it is necessary to find the correlation among the physicochemical properties and biological applications of HAP nanoparticles. With this being said, biorelevant parameters such as shape, size, functional group, morphology, and crystalline phase will determine the surface reactivity/biocompatibility of the nanoparticles [4]. The influence of these factors on biological activity will be discussed in the following sections, as well as the characterization techniques used to study the aforementioned. This review will provide a proper understanding of the physicochemical properties and characterization of HAP nanoparticles that are related to a specific biological response in drug delivery. 

## 2. Physicochemical Properties and Characterization

To employ the appropriate material for a specific application, it is important to determine its chemical, physical, and biological characteristics. In the case of bioceramics, in vitro behavior of bone cells cultured in different samples differs in micro-structural, chemical, and mechanical properties [7]. This section presents an overview of the most important physicochemical characterization techniques for studying HAP nanoparticles. The chemical characterization techniques include Infrared spectroscopy (FTIR) and Raman spectroscopy. In the case of physical characterization, we discuss X-ray Diffraction (XRD), Scanning Electron Microscopy (SEM), Dynamic Light Scattering (DLS), and the Brunauer–Emmett–Teller method (BET).

### 2.1. Chemical Structure and Properties of HAP Nanoparticles

HAP is part of the family of apatites which have the composition M_10_(ZO_4_)_6×2_. The elements M, Z, and X can be occupied by (Ca, Sr, Ba, Cd, Pb), (P, V, As, S, Si, Ge), and (F, Cl, OH, O, Br), respectively. Under biological conditions, HAP usually goes through the substitution of several ions, and the structure can be best described as (Ca, Z)_10_(PO_4_, Y)_6_(OH, X)_2_, where Z = Na^+^, Mg^2+^, K^+^, Sr^2+^, and Y = CO_3_^2−^, HPO_4_^2−^ and X = Cl^−^, F^−^ [10]. In HAP, ion substitution plays a major role in its physicochemical characteristics. For example, ion substitution with CO_3_^2−^ has an impact on its solubility and is also reported to increase the local concentration of Ca^2+^ and PO_4_^3−^ ions to improve bone formation. Moreover, carbonated HAP has improved bioactivity compared with pure HAP, which is attributed to a greater solubility of the carbonated phase [22]. It is also reported that both CO_3_^2−^ and SiO_4_^4−^ replacement on the structure reduce HAP crystallinity [23].

### 2.2. Infrared Spectroscopy

For biomedical research, FTIR is used for studying surface chemical analysis, purity, polymerization kinetics, and the degree of conversion in composites [24,25,26,27]. To perform studies related to HAP, FTIR is relevant because as the bone contains carbonated HAP containing 4–6% carbonate by weight, different compositions and substitutions show different levels of bioactivity [6]. FTIR can be used to determine the type of substitutions within the HAP structure and which ions are being replaced. It is also useful for identifying dopants in biological apatite, which is vital for the synthesis of the final biomaterial. Nanostructured HAP preparation and characterization reported by Fathi et al. was carried out to study its in vitro behavior [6]. The indication of the formation of pure or stoichiometric HAP was confirmed through the presence of characteristic HAP peaks (Table 1). On FTIR spectra, the first indication can be seen at a broadband centered at 1000–1100 cm^−1^. These peaks correspond to the symmetric P-O stretching vibration of the PO_4_^3−^ ion [6]. However, the CO_3_^2−^ vibration mode can be observed at 1465–1415 cm^−1^ and 876 cm^−1^, which suggests that the obtained material corresponds to a carbonated HAP. The type of CO_3_^2−^ substitution can also be determined through FTIR spectra; an A-type CO_3_^2−^ substitution corresponds to the replacement of OH^−^, while B-type CO_3_^2−^ substitution corresponds to PO_4_^3−^ ion. For example, in a study carried out by Segvich et al., bone-like mineral powder and carbonated apatite powders were analyzed, and the CO_3_^2−^ peaks indicated that type-B CO_3_^2−^ substitution had occurred on bone-like material. In contrast, on sintered apatites, the small peak at 1455 cm^−1^ was indicative of type-A CO_3_^2−^ substitution [28]. This information is relevant because the biological response to synthetic apatites depends on the type and degree of carbonation [29], which can be correlated with the availability of hydroxide groups and its electrostatic interactions with cellular molecules, such as proteins, phospholipids, oligosaccharides, to mention some, modulating cellular pathways. Additionally, the intensity ratio between 880 and 872 cm^−1^ bands (I_880_/I_827_) can be used to determine the ratio between CO_3_^2−^ substitutions of OH^−^ (A-type) and PO_4_^3–^ (B-type) groups in carbonated apatite [10], allowing us to determine the solubility or resorbability rate that the HAP nanoparticle could have in an in vitro or in vivo environment. The main vibration frequencies of HAP can be seen in Table 1.

As HAP can be synthesized or obtained from natural sources, the removal of organic components can be carried out with the calcination of the samples, and this is confirmed through FTIR data. In a study performed by Sofronia et al., natural HAP was extracted from a piece of bovine bone and compared with synthetic commercial HAP to study the structural and surface modifications related to the sample’s origin and calcination temperature [30]. In natural HAP, bands associated with collagen and other proteins can be found through a range of amide groups. In Table 1, the intensity of the bands specific to amide groups at 1540, 1418, and 1456 only appears at samples without thermal treatment and calcination at low temperatures. Certain signals can provide us with better knowledge and understanding of the HAP nanoparticles, which will be useful to tailor the biological response of the drug delivery system. The CO_3_^2−^ content can determine the chemical or physical adsorption of proteins and other biomolecules, which is a process dominated by electrostatic interactions. Wang et al. reported that HAP tends to adsorb fibronectin via chemical interactions when containing moderate or almost no CO_3_^2−^ content. On samples with a higher CO_3_^2−^ content, this adsorption can be physical rather than chemical, as there are less adsorption sites [33]. B-type CO_3_^2−^ substitution, which corresponds to the replacement of phosphate groups, is the preferential substitution in bone, which causes a deficit in the negative charge [34]. 

Furthermore, morphology can be influenced by the CO_3_^2−^content, by changing from a needle or rod morphology to a spheroidal morphology [35]. In the biological response, a higher and bioactivity was seen on carbonated HAP due to its enhanced dissolution rate and solubility [36]. For these reasons, CO_3_^2−^ content and other substitutions are highly relevant. The determination of substitutions through FTIR characterization can be complemented with Raman spectroscopy, which will allow us to fingerprint HAP through the vibration of the molecules.

Other anionic substitutions, such as F^−^ and SiO_4_^4−^, can happen in HAP during synthesis, at room temperature, or even body temperature [34]. F^−^ substitution happens at the OH^−^ sites of HAP and leads to a contraction of the unit cell along the *a*-axis due to the smaller radius of F^−^ (0.119 nm) [37]. In addition, due to the greater crystal size, this substitution increases the value of the *c*-axis parameter [37]. The effects on this substitution in terms of thermal stability and mechanical properties are favorable, as 50% F^−^ substitution results in higher thermal stability, the flexural strength of 170 MPa, and the Vickers hardness of 7 GPa [37,38]. F^−^-substituted HAP possesses higher stability in biological environments due to its decreased solubility in acidic conditions. Furthermore, it is more resistant to corrosion in body fluids [34]. Regarding the biological response, F^−^ substitution influences the cell behavior, as a high amount of F^−^ produces low surface potential, favoring cell attachment [39].

This substitution can be identified in the FTIR spectra through the shift and split of the OH stretching vibrations into new bands [40]. The characteristic OH vibration and vibration bands of HAP located at 3570 cm^−1^ and 633 cm^−1^, respectively, are shifted and split into several bands on the FTIR spectra. It is also possible to determine the amount of OH^−^ ions substituted with F^−^ through the identification of a single band present at 3570 cm^−1^ and at 3538 cm^−1^. If the latter is present, at least 75% of the OH^−^ ions are replaced, whereas the band at 3570 cm^−1^ indicates that less than 5% OH^−^ ions are substituted [40]. SiO_4_^4−^ ions can completely or partially substitute for the PO_4_^3−^ ion within the HAP structure. This replacement leads to an increased electronegativity of the surface and a surface charge decrement, which can cause a more negative character for electrostatic interactions [41]. Several reports have acknowledged that SiO_4_^4−^-substituted HAP has an increased bioactivity than that of pure HAP [42,43,44,45]. However, Bohner et al. argued that this improved biological performance cannot necessarily be correlated to the incorporation of Si in the structure, but rather to the increased number of microstructural defects surrounding the grain boundaries [45].

According to Balamurugan et al., the loss of phosphate groups resulting from this substitution can be detected by two additional peaks at 881 and 498 cm^−1^ in the FTIR spectra. Moreover, a decreased intensity in the OH^−^ band at 3570 cm^−1^ strongly suggests that Si^4+^ ions are incorporated within the HAP lattice structure replacing P^5+^ ions [31].

Regarding cationic substitutions, these can occur at two different Ca sites, Ca(I) and Ca(II). The site in which the Ca ion is replaced depends on the ionic radius of the replacing ion. If the ionic radius of the cation is bigger than that of Ca^2+^, the cation tends to be incorporated in the Ca(II) site. These cationic substitutions influence the unit cell volume and may cause a charge imbalance if a monovalent cation occupies the Ca site [34].

### 2.3. Raman Spectroscopy

Distinguishing and identifying different CaP phases is crucial for a proper understanding of their biological response [46]. Raman spectroscopy is commonly used to detect and discern between CaP phases [10]. The most characteristic band (Table 2) for HAP is ν1 (PO_4_), located at 960 cm^−1^, which derives from the asymmetric non-degenerated stretching mode of a free tetrahedral PO_4_^3−^ ion [47,48]. Three additional PO_4_^3−^ peaks are found at 400–500 (ν2), 550–650 (ν3), and 995–1120 (ν4) cm^−1^. The band ν4 at 995–1120 cm^−1^ is key for determining whether HAP or another phase such as octacalcium phosphate is present. Discerning between CaP phases is very relevant because of differences in the ion content. In the case of CaPs, a lower content of OH^−^ and PO_4_^3−^ ions can alter its capacity to form electrostatic interactions. For example, tricalcium phosphate (TCP) has a lower content of OH^−^ groups, whereas HAP has more of these groups, which improves its electrostatic interactions with other molecules. Octacalcium phosphate can be distinguished from HAP through the presence of the ν3 PO_4_^3−^ band centered near 1010 cm^−1^ [47]. TCP can be found through ν1 modes of PO_4_^3−^ in 939, 946, 955, and 968 cm^−1^ [30]. Structural ordering of HAP can also be determined via Raman spectroscopy. Particularly in hydrothermal synthesis, it was found that with an increase in temperature, the bands became narrow and the intensity ratios ν1/ν2, ν3, and ν4 increased [49]. This phenomenon was also seen in a study carried out by Sofronia et al., where different HAP samples treated at higher temperatures exhibited a narrower Raman shift at around 900 cm^−1^ [30]. As previously reported with FTIR spectroscopy, it is possible to discern natural HAP and bone-like structures from synthetic HAP [28,30]. This is achieved through the identification of compounds such as CaF_2_ located around 322 cm^−1^, which can be found in dentine and enamel. However, CO_3_^2−^ can interfere with the measurements of CaF_2_, as the bands are both located close to each other [47,48].

The structure, morphology, and composition of the materials can be determined through physical characterization techniques such as SEM, TEM, and XRD. The determination of these properties is crucial for its biological response, as a different morphology can cause different affinities to biomolecules, and the electrostatic interaction can be modified through the exposure of different crystallographic planes.

### 2.4. X-ray Diffraction

The XRD technique allows one to identify crystallinity, residual stress, texture, and perform phase analysis [52]. Furthermore, changes in crystal parameters regularly induce changes in crystallinity, thermal stability, morphology, solubility, and other physicochemical and biological properties of the biomaterial [2,10], which is essential because small changes in the synthesis temperature and pH can cause differences in the crystallinity and lattice parameters. Characterized by the space group P6_3_/m, HAP’s crystal structure has a *c*-plane with negative charge due to its rich content of PO_4_^3−^ ions and a positively charged *a*-plane due to its high content of Ca^2+^ ions [4]. The presence of unexpected degradation products such as TCP can be demonstrated through XRD [7]. Identifying these forms of CaPs is relevant because they can modify the surface and the level of absorption, affecting cell adhesion and growth [53]. In a study carried out by Rouahi et al., the physicochemical quality of four groups of HAP particles and its influence on protein adsorption and cell response was determined through XRD, showing that even if HAP powders and HAP ceramics (sintered) had highly crystalline similarities with an almost identical XRD diffraction, an inverse relation exists between the specific surface area of HA powders and the protein adsorption capacity, and the cell attachment and protein adsorption on HAP ceramics, a relation based on its physicochemical characteristics [7]. It is also possible to study calcium deficiency on CaPs with XRD analysis, which is carried out through the shift of diffraction peaks. This shift, being proportional to the lattice distortion, can be correlated to calcium deficiency. Moreover, the crystallographic orientation of a given morphology can be studied [10]. The broad peaks in the region between 20 and 50° are characteristic of partially amorphous or nanoparticulate HAP [54] (Table 3). Specific planes, such as (001), are known for being the most thermodynamically stable and the (010) plane is known for being dominant in the morphology of the natural material [55]. The determination of specific planes and phases with characteristics that provide a better interaction between biological systems and biomolecules is extremely important during the design and synthesis of the nanoparticles, as well as the CaP phases present in the final product. The nanoscopic character, appearance of CaP phases, and planes are properties that can be studied through XRD. One example of the influence of different Ca/P composition and CaP phases can be seen in a study carried out by Natesan et al., where a biocompatibility comparison of dicalcium phosphate, TCP, and HAP was performed on fibroblasts. Better cell growth was seen on HAP samples, followed by dicalcium phosphate and TCP. Furthermore, a morphological difference was seen between cells from the CaP samples. On HAP, the cells were flat and constantly expanded to reach neighboring cells, whereas cells from the dicalcium phosphate sample were circular. In this study, TCP exhibited the least compatibility. The authors mentioned that a better biocompatibility may be due to the presence of an additional OH^−^ group in HAP, which is known for promoting cell growth. On the other hand, TCP, due to its lack of OH^−^ ions in the structure showed a limited growth of cells [56]. In the case of the crystal orientations, the *a*,*b*-plane and *c*-plane have different functions, as they are seen in response in different parts of the body. For example, the *a*,*b*-plane is bioactive for human saliva and bioinert for the surface of human teeth. For this reason, an HAP surface with *a*,*b*-plane would be suitable in a bone filler [57].

### 2.5. Scanning Electron Microscopy

**Figure 1:**
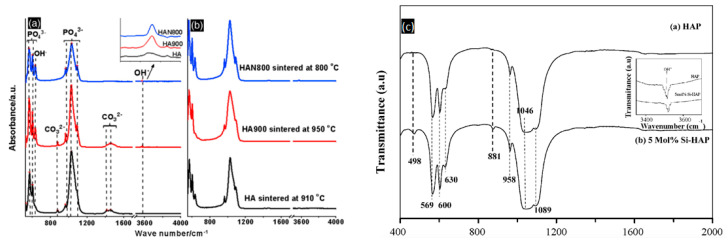
(**a**) FTIR spectra of HAP. Reproduced with permission from [30], Elsevier, 2014. (**b**) FTIR spectra of Si-substituted HAP. Reproduced with permission from [31], Elsevier, 2008. (**c**) FT-IR spectra of (a) pure HAP and (b) 5 mol% Si-substituted HAP.

**Figure 2:**
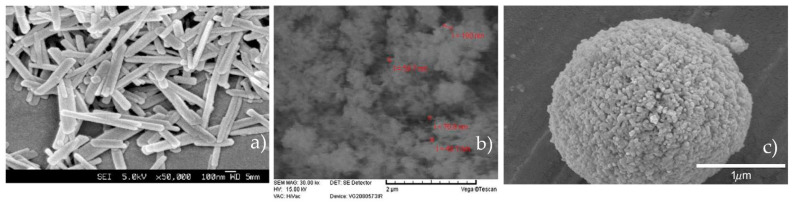
(**a**) SEM micrograph of rod-like nanocrystal HAP synthesized using cationic surfactants. Reproduced with permission from [59], The Royal Society of Chemistry, 2003. (**b**) SEM micrograph of the synthesized HAP via chemical precipitation. Reproduced with permission from [14], Elsevier, 2008. (**c**) SEM micrographs of spherical HAP nanoparticles synthesized via ultrasonic spray pyrolytic processing. Reproduced with permission from [62], Elsevier, 2009.

**Figure 3:**
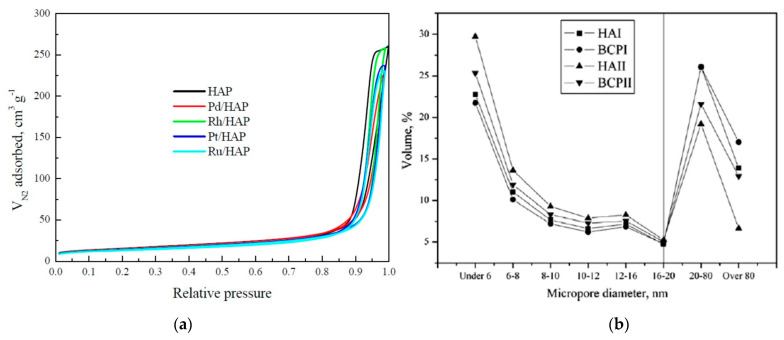
(**a**) N_2_ physisorption isotherms for the prepared catalysts. Reproduced from [72], MDPI, 2021. (**b**) Size distributions of micropores on the four types of CaP particles. Reproduced with permission from [74], Elsevier, 2010.

Morphology plays an important role in the design and application of different HAP particles. Different HAP morphologies can be obtained by using different synthesis techniques, and the SEM technique gives, at a nanoscale level, plenty of accessible data about the nanoparticle’s characteristics, such as particle size, morphology, and porosity. Rod-like HAP nanoparticles have been used recently and taken into consideration because natural bone is an inorganic material with nanocrystalline rod-like structures [21]. Moreover, it has been found through cell culture experiments that the incorporation of rod-like nano-HAP on scaffolds results in higher biocompatibility, bioactivity, and mechanical properties [58]. In a study carried out by Yao et al., rod-like HAP nanoparticles ranging from 500 to 1000 nm were obtained by using a cationic surfactant as a template to form the micelles for the nucleation sites of HAP. The authors explain that one possible mechanism of rod structure formation stems from micelles formed by hexadecyl(cetyl) trimethyl ammonium bromide -PO_4_^3−^ mixtures. As the PO_4_^3−^ groups are located on the surface of the micelles along with the presence of Ca^2+^ ions, Ca_6_(PO_4_)_6_ clusters are preferentially condensed on the rod-shaped micellar surface. This happens due to higher conformation compatibility between the hexagonal shapes of the micelles and the Ca_6_(PO_4_)_6_ clusters [59]. The SEM micrograph of the rod-like HAP nanoparticles can be seen on Figure 1. Eslami et al. observed needle-like agglomerates of nanostructured HAP obtained through chemical precipitation (Figure 2). As the synthesis of HAP was carried out in an alkaline environment, the highly corrosive OH^−^ groups caused holes and structural defects in the structure [14]. In the same study, it was also shown that with the addition of F^−^ ions on the structure, the aspect ratio of the crystallites significantly decreased, and fewer agglomerates were formed compared to HAP alone [14]. With this being said, the addition of different compounds during the synthesis process and the environment in which it takes place can affect the final morphology of the HAP nanoparticles. The formation of HAP can be divided into homogeneous nucleation, the aggregation of primary amorphous CaP particles into typically spherical units, the aggregation of spheres into chain-like structures, the growth of these structures, secondary precipitation, and phase transformation [10]. This aggregation model is consistent, as seen in Figure 3. Spherical nanoparticles can also be obtained using anionic surfactants such as sodium lauryl sulfate [60]. Regarding the concentration of surfactant, a trend has been found where at higher concentrations, spherical nanoparticles with diameters of 100 nm can be obtained, whereas using lower concentrations results in rod-like nanoparticles [60]. One can achieve a better interaction via Van der Waals forces by using rod HAP nanoparticles due to their higher superficial area [21]. It would be reasonable to say that adding anionic or cationic surfactants defines the final shape of the nanoparticle due to the micelles that are formed. However, HAP usually grows along the *c*-axis because of the phosphate groups and the cell parameters, which results in a rod nanoparticle [61]. The effect of nanoparticle morphology and shape on the biological response is discussed in further sections.

### 2.6. Differential Scanning Calorimetry

Differential Scanning Calorimetry (DSC) can be used to study the thermal stability and decomposition of HAP. It has been reported that for carbonated HAP, the decomposition temperature is reduced as low as 700 °C compared to non-carbonated HAP [63]. In HAP specifically, heat treatment plays a major role in pore size and crystallinity. Calcination treatments are known for narrowing the pore size distribution and the enlargement of particle size distribution [30]. Generally, mesoporous structures are formed by a self-assembly process governed by non-covalent forces such as hydrogen bonds, Van der Waals forces, and electrostatic interactions between surfactants and ceramics. [64] During the synthesis process, once the HAP source has condensed around the micelles, the surfactants are removed by solvent extraction or calcination, resulting in a mesoporous structure. The pore size can be studied by thermoporometry, a technique based on the thermodynamic relationship between the pore size and the melting temperature of the water within them [65,66]. The most important advantage of this technique is that sample preparation is extremely straightforward. For the calculation of pore size, the Gibbs Thomson formula is used, considering the densities of the materials. These studies concluded that measuring pore size through thermoporometry is easier, but it would be adequate to use more robust methods, such as the nitrogen desorption technique or the Brunauer–Emmett–Teller (BET) method. 

### 2.7. Brunauer–Emmett–Teller Method

The importance of structural properties in the design of delivery systems demands the characterization of various characteristics exhibited by porous solids. Some standard methods used in HAP characterization include liquid intrusion, SEM, and X-ray and neutron scattering. Nevertheless, gas adsorption is the conventional technique for mesoporous materials due to its optimal pore-size measurement ranges, which are found to be between 1 nm and 20 nm. Experimental adsorption results are then evaluated by the well-established method of Brunauer–Emmett–Teller (BET) [14]. As previously mentioned, gas adsorption is usually implemented to characterize HAP mesoporous materials due to its optimal pore-size measurement ranges, which are found to be between 1 nm and 20 nm. Several groups have previously characterized HAP materials using the BET method [28,32,67,68]. By studying the amount and size of pores, one can tailor the biocompatibility, as was observed in a study carried out by Natesan et al., where a trend was observed on materials that exhibited pores. Fibroblasts thrived on porous HAP, as they appeared to be flat and make contact with nearby cells, as well as infiltrate through pores. HAP and dicalcium phosphate samples exhibited pores of around 50–60 µm, whereas TCP had no visible pores [56]. For cells to have a good growth and development on a porous structure, it is known that pores should have a maximum size of 100 µm. In addition to pore size, BET is also helpful for determining the type of porosity of a material. For example, as mesoporous HAP was used for the delivery of doxorubicin and vancomycin [8,55,68]; the mesoporous nature of the nanoparticles can be determined through the types of isotherms from adsorption–desorption studies. Mesoporous materials usually exhibit a type IV or I isotherm and the shape of the graph is associated with the process of capillary condensation, which usually occurs on pores within the size range of 2 and 50 nm [69]. Although porosity has been shown to improve cell adhesion in osteoclasts [56], the main disadvantage of these platforms would be that extremely small pores do not have the ability to adsorb large amount of biomolecules with large molecular weights. In this regard, mesoporous HAP can be synthesized with larger pores of about 10–12 nm to design drug delivery systems [67]. BET is also helpful for the determination of the surface area of HAP nanoparticles. It has been reported that it is possible to control the surface area through the calcination temperature. In a study by Dasgupta et al.*,* there was a decrease in the surface area as the calcination temperature was higher. With a change in temperature from 600 °C to 800 °C, the surface area changed from 73 to 57 m^2^g^−1^ [19]. Nanoparticles with large surface areas can be achieved by using nonionic surfactant emulsion. Specifically, Bose and Saha synthesized HAP nanopowders with surface areas as high as 130 m^2^g^−1^ with needle and spherical morphology using the reverse micelle-processing route [70] Even higher surface areas can be achieved through a mixed-surfactant template. Uota et al. reported the synthesis of HAP nanoparticles encapsulated with calcium stearate using polyethylene (20) sorbitan monostearate (Tween 60) and nonaethyleneglycol monododecyl ether (C12EO9) with resulting surface areas of 362 m^2^g^−1^ [71]. 

As shown in Figure 3a, in a study by Boukha et al. a superficial area between 48–55 m^2^ g^−1^ was found in a comparative study with doped apatites of noble metals [72]. Curves showed type V isotherms, all samples had similar shapes and hysteresis loops as usual in mesoporous solids proceeding through a monolayer and multilayer hysteresis followed by capillary condensation. Synthetic apatites without the used surfactants tend to have low superficial area and porosity due to high sinterization temperatures compared to those biological apatites ranging from 100 to 200 m^2^ g^−1^ [73]. As shown in Figure 3b, Zhu et al. observed the microstructure in biphasic calcium phosphate, and found larger micro porosities than those in apatites powder, with particle distributions ranging from 2 to 200 nm [74]; those samples showed a higher protein adsorption.

### 2.8. Dynamic Light Scattering

Dynamic Light Scattering (DLS) is used in the field of biomaterials and nanotechnology for the determination of the hydrodynamic size of nanoparticles, and it can also measure the resultant surface charge with the help of an electrode [75]. For the characterization of HAP nanoparticles, several authors have used this technique for the study of particle size and zeta potential [30,59,76]. The stability of colloidal systems can be determined by zeta potential. This parameter decides the magnitude of the electrostatic forces between particles and influence their aggregation. Particularly, increasing its absolute value enhances the electrostatic repulsion, providing higher stability to the particles in suspension [77]. Besides particle size determination, DLS allows for the determination of the surface charge of nanoparticles through zeta potential. The values of different surface charges from modified or functionalized HAP nanoparticles is essential for the specific interaction of positive, negative or neutral molecules. The surface charge of HAP nanoparticles can influence its cellular uptake as well as the behavior of the cells [78]. In a study by Frankenberg et al., HAP nanoparticles with a positive charge were more easily taken up by MC3T3-E1 cells than other nanoparticles with similar sizes. This was due to the electrostatic attraction between the positive charge of HAP and the negative cell membrane [78].

## 3. Interactions in Drug Delivery with HAP Nanoparticles

Recently, HAP nanoparticles have been used as vehicles for delivery due to their affinity to DNA, proteins, several drugs, and proper release activity (Table 4) [79]. Although there are no conclusions on which HAP nanostructure is more suitable for which kind of molecule, the adsorbed amount is a function of the functional groups interactions, surface area, porosity, pH, and the surrounding medium [18,80]. Regarding the porosity and pore structure effect on nanoparticles’ ability to adsorb and retain the cargo, it is known that mesoporous inorganic materials with high pore volume and adequate pore size are able to adsorb higher amounts of therapeutic molecules and ensure a sustained release [18,74]. Although the synthesis process influences pore size and structure, factors such as the amount and size of the pores are primarily a function of the composition of the raw materials and the sintering conditions. A sintering process up to 1350 °C maintains the HAP phase while promoting pore formations [80]. However, sintering procedures at higher temperatures makes other phases such as TCP appear due to HAP decomposition. This can be observed in Table 2, where the TCP band appears at a sintering temperature of 1400 °C in a study by Sofronia et al. [30]. Regarding the effect of particle size, it is known that for solid drug delivery systems, it has a strong impact on its dissolution and drug absorption. Given the large surface area of nanoparticles, an increase in the bioavailability of poorly soluble drugs can be seen in several studies [81,82,83]. A study to correlate the influence of particle size specifically with HAP was performed by Rouahi et al., where they found that HAP nanopowders containing 100 nm particles adsorbed more proteins than 1 µm [7]. This was directly attributed to the difference in superficial surface area, where smaller nanoparticles had higher superficial surface areas than micro-scale HAP particles. The results of these studies suggest that a higher superficial surface area leads to higher protein adsorption, which is also reported in other studies [84]. 

As mentioned above, particle size, porosity, and pore size determine the scale of the surface area that interacts with proteins, DNA, and drugs. The control of pore size/distribution, porosity, and particle size regulates the surface area, as higher porosity leads to higher surface area [84]. Hence, a high surface area allows proteins to interact with the surface and bond or interact through electrostatic forces and hydrophobic interactions [84]. Besides the aforementioned properties, the physical and chemical interactions mainly define the ability to retain cargo. The conjugation of therapeutic agents or biomolecules to the nanoparticles can be classified into two major groups: (1) conjugation through covalent linkages and (2) attachment through physical interactions [18]. The identity of the cargo determines the approach and the kind of interactions involved in the conjugation. Nevertheless, it is generally known that the chemical nature of CaPs induces protein interactions through electrostatic or hydrophobic interactions, and more efforts have been made to clarify the role of solubility, degradation rate, and zeta potential because these regulate the charge density, also known as adsorption sites [84]. Each type of therapeutic agent or biomolecule is discussed separately to understand the importance of these characteristics. 

### 3.1. Proteins

As it is essential to tailor the characteristics of HAP nanoparticles to control the affinity of the cargo with the delivery system, several authors have studied the relationship between different physicochemical properties and protein absorption on the surface [44,52,55,56]. CaPs are able to adsorb more protein than other materials, as calcium and phosphate ions are present as preferential binding sites for proteins. Several authors studied the protein adsorption potential of HAP powders treated with heat, where it was found that there are two main correlations between the superficial surface area and the protein adsorption capacity of HAP: the higher the superficial surface area, the higher the protein adsorption; however, when HAP is sintered, intergranular microporosity is formed and less proteins can be adsorbed [7,28]. In a study by Rouahi et al., an HAP powder with agglomerated granules and a low value of surface area due to the partial fusion of the particles was synthesized. For the FTIR characterization, although the HAP composition was confirmed, the formation of TCP was not observed even though the sample was treated with high heat, which is contrary to other studies displayed in Table 1, where Sofronia et al. obtained TCP after sintering at 1400 °C [30]. However, the carbonate peak located around 1500 cm^−1^ disappeared after the heat treatment. Nevertheless, the authors explained that the heat treatment did affect the protein adsorption potential and that the slight difference between samples was due to the surface area values, where the original samples that were not sintered had higher surface area values than those that underwent heat treatment. Finally, the ceramics prepared from the sintered samples had higher microporosity and intergranular microporosity, which explained the higher values of cells attaching on the surface. According to the previous study, increasing the surface area results in higher protein adsorption. Furthermore, as the microporosity decreases, the lower the protein adsorption and cell attachment rates become [30]. 

Another study that discussed the relationship between the calcination temperature and the adsorption and controlled release of bovine serum albumin (BSA) was carried out by Dasgupta et al. [19]. The authors reported a process of synthesis of nano-HAP using a reverse micelle template system and protein loading through electrostatic interactions between the nanoparticle surface and the charged amino acids of BSA. The release profiles were evaluated at three different pH solutions. During the physicochemical characterization, when the particles were calcined at 600 °C, pure HAP was formed instead of TCP, which forms at higher temperatures and is explained by the absence of the OH^−^ bending frequency in FTIR spectra. This phase transformation happening at higher temperatures was noticed because of the disappearance of the HPO_4_^2−^ at 870 cm^−1^ and the OH^−^ band at 3569 cm^−1^. The authors also mentioned that this change was reflected in the XRD pattern [19]. In the case of particle size analysis, it was found that at higher calcination temperatures, the particle size increased as well. The HAP nanocrystals calcined at 600 °C showed the highest surface area (73 m^2^g^−1^) and aspect ratio. For the loading experiments, those studied at a pH higher than 7.5 maintained high stability because at a lower pH, the dissolution of nano-HAP could destroy the stable interface between the BSA and the nanoparticles. This interface was formed by the positive calcium ions and the negative polar heads of the BSA molecule. Since the pH of the BSA and CaPs suspension was above the isoelectric point of each BSA, TCP, and HAP, both the BSA and the nano-HAP carried negative charges on their surface. The stern layer of anions [A-, (H_2_PO_4_^−^, OH^−^] attached to Ca^2+^ ions was the source of negative charges on the CaP nanoparticle surface. This interaction gradually decreased as the pH increased from 7.5 to 8.5 and 9, due to an enhanced electrostatic repulsion force between the particle surface and the BSA. The surface area was the main factor of interaction, as the higher the surface area, higher the surface charge density of the nanoparticles, resulting in a higher degree of electrostatic interactions. Since the surface of HAP materials cannot be easily modified through surface treatments to form hydroxyl-, amino-, or carboxyl- groups as is possible with metals and polymers, peptides can be adsorbed on HAP by modifying the surface [28]. This was also the case in a study by Kojima et al., where several peptide-HAP complexes were made to adsorb cytochrome c, myoglobin, and BSA [32]. Firstly, the complexes were made by adding peptides during the HAP synthesis process along with the calcium ions source. The peptides contained amino groups on their structures (glutamic acid and lysine) and were easily detected through FTIR spectroscopy, where two major bands of amide I and amide II stretching groups appeared at 1650 and 1560 cm^−1^, signals that according to Table 1 are not found among other HAP examples. Then, selective adsorption was proven with a lysine–HAP complex and the acidic protein BSA. The authors report that the selective adsorption was due to electrostatic interactions between the peptides on HAP surface and the proteins, as adding the peptides changed the surface potential [32]. As carbonate ions can be found in HAP samples sintered at low temperatures, the authors did not discuss whether the small signal they found at 1650 cm^−1^ was due to a possible carbonate substitution on the HAP structure. Even though it is not discussed in the paper, as the signal is smaller than those HAP samples with high carbonate content, it can be assumed that the complex was made between the peptide and HAP. As seen in Table 1, increasing the sintering temperature can reduce the carbonate concentration, thus affecting peptide and protein adsorption as there are less ions for the biomolecules to interact with [85]. In the case of the study by Kojima et al., protein and peptide adsorption was still possible because of the electrostatic interactions between the side chains of the peptide and the proteins [32]. For these reasons, it is important to take into consideration which groups are meant to interact via electrostatic forces and set an appropriate pH to set the expected charges of each groups.

### 3.2. Peptides

Peptides are smaller, cheaper to produce, less susceptible to degradation, and have advantages over proteins [28]. Even if proteins and peptides are both able to promote different cell functions, such as cell adhesion, nanoparticle-mediated peptide delivery is known to enhance the bioavailability of these proteinaceous compounds [24] and protect the peptides from enzymatic degradation [86,87,88,89]. As mentioned in previous sections, the level of peptide adsorption on the nanoparticles can be altered through the presence of carbonate within the structure and changes in surface morphology. In work by Segvich et al., different peptide sequences with preferential adsorption towards HAP, carbonated apatites, and bone-like minerals were identified, and they studied how surface morphology changes and carbonate incorporation could alter peptide adsorption [28]. According to data reported by Tamerler and Sarikaya [90], three 7-mer peptide sequences are specific to HAP, and adsorption experiments were carried out to prove this preferential adsorption. Peptides APWHLSSQYSRT [A], SLTPIPHEFSRE [S], and VTKHLNQISQSY [V] were identified with phage display, and peptide EEEEEEEPRGDT [E] was known to be found in the bone sialoprotein [91]. Peptide E can be found in proteins that contain a high proportion of acidic amino acids, such as glutamic and aspartic acids, whereas peptides A, S, and V do not present strings of acidic amino acids. It is reported that compounds with acidic groups are adsorbed on HAP surfaces due to the interaction with calcium surface cations [92]. The BET surface areas for bone-like material, HAP, 5% carbonate apatite (CA5) and 10% carbonate apatite (CA10) were 121.55, 0.05, 0.11, and 0.19 m^2^g^−1^, respectively. Regarding surface feature sizes, HAP, CA5, and CA10 had granular surfaces, whereas bone-like material illustrated more evident plate-like features. The FTIR characterization showed characteristic type A carbonate peaks at 1455 cm^−1^ for CA5 and CA10 but not for HAP. For XRD characterization, the narrow and distinct signals, because of the high sintering temperature peaks were correlated with HAP. Peaks from TCP phase were found on the CA5 and CA10 samples at around 29.7 and 37.3º, which were also not present on the HAP sample. It was seen that at the same morphological scale, the carbonate concentration differences were less than 2 wt%, which indicated that the differences between peptide adsorption were present due to morphological differences rather than compositional, which is a known phenomenon as reported in other studies [84]. The authors mentioned that a better control on the surface morphology could determine whether carbonate incorporation had an effect on peptide adsorption. In the case of the effect of the peptides’ pH and the amount of acidic amino acids on the structure, as this study demonstrated that peptides without acidic amino acids showed preferential adsorption towards HAP even though it was reported that acidic peptides had a higher affinity, based on the electrostatic attraction, the acidic proteins should preferably be adsorbed on the calcium site- based surfaces, basic proteins preferentially adsorbed on the P/OH site-based surfaces; acidic residues preferably bonded to the Ca^2+^ sites, basic residues preferentially bonded to the P/OH sites [84].

In another study, computational studies were made to understand the interaction between the tripeptide HYP-PRO-GLY with HAP. This peptide is known for being present in collagen protein and might have a growth-modifying effect on HAP surfaces. This peptide contains hydrophilic and hydrophobic side groups, and the results showed that it interacts mainly with the (0110) plane rather than the (001) plane. The main interactions found in this study were located on the surface calcium ions; these were more pronounced than the more thermodynamically stable (001) plane [93]. On another study, peptides HYP-PRO-GLY, PRO-HYP-GLY, PRO-LYS-GLY, and PRO-HYL-GLY interactions with HAP (0001) and (0110) surfaces were studied through molecular dynamics [55]. The four peptides adsorbed strongly to (0110), showing a proton transfer from the peptides to the reactive surface. On the (0001) surface, this transfer only happened when the amino acid residue had a charged polar group (PRO-LYS- GLY and PRO-HYL-GLY). In this particular case, the proton of the LYS and HYL amine group migrated to the basic phosphate group. With these studies, the authors showed that peptide adsorption onto HAP depends on the crystallographic phases [55]. Inhibited crystal growth of HAP has been attributed to the molecular adsorption of certain amino acids on the surface at active growth sites due to the affinity and the nature of each amino acid [94,95,96]. It is reported that amino acids with polar uncharged side groups have a high adsorption affinity for the surface of HAP, which is also the case for aspartic acid, phosphoserine, histidine, and imidazole derivatives [97].

### 3.3. Drugs

Different types of HAP nanoparticles have been used for the delivery of several drug molecules, composites, coatings, and paramagnetic particles. Abbasi Aval et al. developed superparamagnetic HAP-coated Fe_2_O_3_ nanoparticles, aiming to prevent the agglomeration and oxidation of superparamagnetic particles with the coating [67]. The authors synthesized mesoporous HAP with 12 nm sized pores and a surface area of 148 m^2^g^−1^ to adsorb a large amount of doxorubicin, a small hydrophobic drug, on the surface of Fe_2_O_3_ nanoparticles. The nature of the pores was determined by nitrogen sorption isotherms, where, due to a relatively sharp slope of the adsorption–desorption diagram, the presence of cylindrical pores with open ends was confirmed. Under neutral conditions, the positive nature of the HAP surface is caused by the specific adsorption of excess calcium ions and their solubility [92]. The amount of doxorubicin adsorbed on the surface was almost 93%; however, the authors do not mention in which solution or conditions, and the release profiles were studied at pHs 5.5 and 7.4. Within 24 h, in pH 7.4, only 10% of the loaded doxorubicin was released, whereas in the pH 5.5 environment, about 70% of the drug was released, this means that HAP and doxorubicin had a higher affinity when HAP carried positive charges rather than negative [98]. This conjugation with doxorubicin was also seen in another study by Yang et al., with a different morphological approach [8]. The authors synthesized hollow mesoporous HAP nanoparticles with a surface area of 163.2 m^2^g^−1^ and a pore size of 3.3 nm. The samples were placed in phosphate buffer at pH 7.4, and the vehicles showed a fast release for the first 30 min and slow release from 0.5 to 50 h, which corresponds to a pseudo-first-order release profile. They also mentioned that due to this behavior, it could be assumed there was no interaction between the HAP matrix and the doxorubicin molecules, and the release was significantly higher on mesoporous hollow nanoparticles than normal nanoparticles and higher at pH 4.5 than pH 7.4. However, in a study by Storm et al., doxorubicin was encapsulated in higher amounts on negatively charged liposomes than on neutral ones [99]. The main mechanism of adsorption of doxorubicin, in this case, may not be caused by electrostatic interactions but rather hydrogen bond interactions between the OH^−^ group of doxorubicin and the OH^−^ group of HAP. This is reasonable because, in a study by Yulia et al., by combining quantum chemistry calculations and spectroscopic techniques, an ibuprofen/nanoHAP complex was studied [54]. The authors reported that the main interactions of the system were the hydrogen bonds between both OH^−^ groups of HAP and ibuprofen and a strong interaction between ibuprofen’s carbonyl group and the Ca^2+^ center of HAP [54]. Moreover, even though it has been proved that electrostatic interactions play a major role in adsorption onto HAP surfaces, it does not fully account for the adsorption of biomolecules. For example, the adsorption of human serum albumin occurs under conditions where the adsorbent and the adsorbate are negatively charged. This process was dominated by entropy, structural rearrangements, changes of hydration, and co-adsorption of electrolytes. Moreover, studies suggest that the secondary structure of the biomolecules and desorption of water also play critical roles [100,101].

In a study by Barroug and Glimcher, the anti-tumor drug cisplatin was adsorbed by HAP crystals of 93 × 29 nm [102]. The authors mentioned the effect of the solution’s composition and ionic strength, in which an increase in the ionic strength of the solution significantly reduced the affinity between the HAP surface and the cisplatin molecules. This dependence of adsorption on the solution composition is driven by electrostatic interactions, as the surface is covered by adsorbate, and charge neutralization and adsorbate–absorbate repulsion occur as well. In these experiments, the HAP samples synthesized at a pH close to 10 had isoelectric points at around 7.0, and the highest uptakes were observed after equilibrium with phosphate buffers rather than Tris buffers due to the presence of phosphate ions, which can also be explained by the hydrolyses of cisplatin in aqueous solutions [102]. The authors also reported that under the conditions of the experiments (pH 7.4, phosphate 10 mM), the HAP crystals and the cisplatin were oppositely charged, which resulted in an electrostatic attraction between both surfaces. With this being said, the medium in which the adsorption between HAP and the drug occurs plays a major role in its performance and release, as the hydrated derivatives and the presence of different ions can cause displacements in the native forms of the drugs.

As the structure of HAP contains negatively charged OH^−^ groups, these can interact with positive groups such as amine groups, sodium, and hydrogen ions. These interactions were employed in a drug delivery system using HAP and sodium ampicillin. In a study by Queiroz et al., they made a comparison between HAP and HAP composites that contained other crystalline phases such as TCP. The authors explained that pure HAP adsorbed more ampicillin than the composites with 16 and 57 wt% TCP because of a greater amount of OH^−^ groups in HAP, which are bridging agents to ampicillin. The higher solubility of TCP also played a major role in decreasing the ampicillin adsorbed, causing ampicillin resorption during the loading process, making it harder for ampicillin to adsorb on the composite surfaces compared to the HAP sample, which was relatively insoluble [103].

### 3.4. Genetic Material

HAP can be used as a vector for gene delivery due to their strong affinity and the ionic interactions between calcium ions and the gene backbone [104]. This allows the use of HAP delivery systems for the attachment of regulatory sequences and movement across the cell membrane. One of the main disadvantages of using HAP as a delivery system is that the sintering process can cause the agglomeration of particles, which in the case of gene delivery decreases its transfection efficacy. In a study by Han et al., well-dispersed HAP nanoparticles were obtained by a simple ultrasound-assisted precipitation method with the assistance of glycosaminoglycans [77]. The nanocrystalline nature of the particles was confirmed by the broadening and merge of the three major peaks (211), (112), and (300) at around 2θ = 30, which are characteristic of HAP. They were also able to confirm the presence of carbonate ions due to the peaks at 603 and 567 cm^−1^, which are phosphate bands appearing in different sites [77]. The size for the rod-like particles was about 20 × 50 nm and had a zeta potential of −60.9 mV, which improved stability, as mentioned in previous sections. The authors also mentioned that the acoustic cavitation caused by the ultrasound processing dispersed the HAP nanoparticles. The addition of glycosaminoglycans improved the electrostatic interaction between their negatively charged groups and the calcium ions of HAP, resulting in the overall negative charge of HAP nanoparticles. A novel strategy for gene therapy involves the use of biominerals through the nucleation of HAP on a DNA template. The rationale behind this stems from the relation between DNA and HAP in other biological systems and a strong interaction between both materials. As a specific binding activity of HAP exists for DNA, these kinds of complexes are less susceptible to degradation by serum and nucleases [105]. In a study by Bertran et al., DNA was encapsulated into HAP nanoparticles through the fabrication of nanocapsules and crystalline nanorods with DNA inside. The experiments suggested that HAP grew around the DNA matrix [105].

**Table 4:** Drug delivery applications for HAP and its respective cargos.

	Cargo	Heat Treatment		Size	Potential	SSA	Porosity	Pore Volume	Morphology	Amount Adsorbed	Application	Reference
		(°C)		(nm)	(mV )	(m^2^g^−1^)	(%)	(cm^3^g^−1^)	(-)	(mg)		
	Fibrinogen				-	2.53	2.39	-	Spheres	2.93 mg/m^2^		
	Insulin	80 overnight		60						2.24 mg/m^2^	Diabetes	[74]
	Col-I									1.12 mg/m^2^		
	BSA	1250	4 h	1000	−37	0.9	micropores	-	Granules	65.7 µg/mL	-	[7]
	BSA	1000	15 h	100	0	25.4	micropores	-	Granules	78.3 µg/mL		

	BSA	600	3 h	39	−0.55	40	-	-	-	4.0 mg/m^2^		
Proteins	MG	600	3 h	39	−0.55	40	-	-	-	1.0 µg/m^2^	Blood compatibility	[85]
	BSA	700	3 h	43	−0.9	20	-	-	-	9.8 mg/m^2^		
	MG	700	3 h	43	−0.9	20	-	-	-	1.5 µg/m^2^		
	BSA	600	4 h	32	-	73	-	-		89 µg/mg	Delivery	[19]
	BSA	700	4 h	36	-	66	-	-		85 µg/mg		
	
	Cyt c	60	3 h	60 × 30	−24	96		0.79	Rod	60 µg/mg		
	MGB									43 µg/mg	Delivery	[32]
	BSA									78 µg/mg		
Peptides	APWHLSSQYSRT	1350	1 h	-	-	0.05	-	-	Granules	1 nmol		
	STLPIPHEFSRE			-	-					2.4 nmol	Delivery	[28]
	VTKHLNQISQSY			-	-					2.5 nmol		
Drugs	Doxorubicin	100	24 h	400 × 600	-	163.2	mesopores	0.53	Oval	3 × 10 ^5^ mol/g	Breast cancer	[8]
	Ibuprofen	1000	2 h	79	-	-	-	-	Plates	-	Arthritis	[54]
	Cisplatin	80		93 × 29	-	96.8	-	-	Plates	2.4 mg/g	Cancer	[102]
	Ampicilin	1200	1 h	8–9 ×10^3^	-	-	Mesopores		Spheres	6.5 mg/g	Bacterial infection	[103]
DNA	Fish sperm DNA	80	1.5 h	20	-	-	-	-	Spheres	11 µg/mg	Gene therapy	[105]
	EGFP-N1 pDNA	170	2 h	40–60	-	-	-	-	Rod	0.02 µg/ug	Gene therapy	[106]
	CDglyTK	35	72 h	23–34	+16.8	-	-	-	Feather	-	Antitumor	[107]

SSA: superficial surface area, Col-I: type I collagen, BSA: bovine serum albumin, MG: macroglobulin, Cyt *c*: cytochrome *c*, MGB: myoglobin.

## 4. Intracellular Activity

A carrier’s ability to deliver the cargo depends on the cellular uptake process. Hence, understanding the interactions between nanoparticles and cells is necessary for the proper design of the delivery system [108]. Furthermore, it is necessary to consider several requirements to ensure its safe use in vitro and in vivo. Some of these requirements include biocompatibility, which should promote cell migration and the transfer of growth and biochemical factors, and biodegradability, as the materials used should be easily removed from the body and not affect other organs [80]. In the case of morphology design, it has been found that the spherical and rod-like shapes of HAP nanoparticles showed remarkably less cytotoxicity, as compared with the needle and plate-like shapes [4]. It has also been observed that rod-shaped nanoparticles have high cell internalization rates and longer blood circulation than spherical nanoparticles [18,109]. Particle size is also an essential factor in the biological response. Particles with a diameter longer than 500 nm are affected by gravitational forces, causing their margination towards the wall [110]. On the other hand, smaller particles (less than 500 nm in diameter) are localized towards the wall due to Brownian motion [111]. CaP and ceramic nanoparticles are widely used for drug delivery applications. However, inorganic nanoparticles do not degrade rapidly and fully in vivo. Hence, many accumulate in body tissues [24]. For this reason, it is crucial to understand the toxicological issues related with nanoparticle-mediated delivery. Compared to HAP bulk ceramics, HAP nanoparticles have exhibited a better osteogenic activity due to their higher surface activity and similarity to bone mineral [112]. In a study by Fulgione et al., biomimetic plate-like HAP nanocrystals with a particle size of 300 nm were synthesized via chemical precipitation [113]. The surface area of the crystals (110 m^2^g^−1^) was close to that of biological HAP nanocrystals (110 m^2^g^−1^). They mention that the HAP used for the study was structurally very similar to bone-like structures due to the presence of carbonate ions and the degree of crystallinity; however, they do not show these data. These nanocrystals were complexed with phage (SR φ1) from *S. Rissen*, which is a Salmonella bacteriophage used to test its performance to control Salmonella bacterial infection. The HAP–SRφ1 complex allowed phage entrance to eukaryotic cells and was stable at very low pH [113].

Different sintering procedures can result in different biological responses and physicochemical properties. In a study by Chen et al., different nano and submicron-sized grains were obtained with different osteoclasts activity in vivo and in vitro [114]. In the case of cell viability, there was no significant difference between the groups. However, on day 3, the highest viability was found in the sample 1 (750 °C for 2 h, 1050 °C for 6 min, heating rate of 150 °C/min), which had a grain size of 87.50 nm, and the lowest viability on sample 2 (heating to 1050 °C with a rate of 10 °C/min, 950 °C for 2 h), which had a grain size of 555 nm, at day 5. In sample 1, the reduction in size to the nanoscale range increased the hydrophilicity and surface energy, and the osteoclasts exhibited a smaller and less defined actin structure. In another study by Yurong Cai et al., HAP nanoparticles with controllable sizes were obtained based on the theory of critical micelle concentration. By adding 6.0 × 10^−4^, 9.0 × 10^−4^ or 12.0 × 10^−4^ M of hexadecyl(cetyl) trimethyl ammonium bromide during the synthesis process, nanoparticles of 20, 40 and 80 nm (np20, np40, np80) were obtained. Mesenchymal stem cells were cultured on the HAP films and the one that exhibited the most rapid cell growth was the np20 film. The nanoparticles had a completely different effect on an osteosarcoma-derived cell line, where it was found that the proliferation rate was slowed remarkably compared with the control. In this experiment, cell proliferation was decreased in the film with np20 [115]. The authors explain that the mechanisms for the selectivity of the effects of nanoparticles on different cell types are not known. However, they suggest that the dissolution of HAP nanoparticles increases the concentrations of calcium and phosphate in the culture medium, altering the gene expression alkali phosphate and proteins [115].

## 5. Concluding Remarks

HAP nanoparticles have been implemented in biomaterials science and tissue engineering due to their biocompatibility and potential as a path for medical treatments. Nowadays, HAP nanoparticles’ design have been mostly focused on the understanding of particle size, morphology, specific surface area, polydispersity, composition, and the physical and chemical interactions involved in the use of HAP nanoparticles as drug, gene, and/or protein/peptide delivery vehicles, neglecting its final goal to improve or restore a cell, tissue and/or an organ function, and putting aside the integral understanding of the environment where these particles will be applied.

In this review, we proposed an initial overview of the fundamental properties of HAP nanoparticles and the characterization of physicochemical properties involved as a delivery system and in their biological responses. From the chemical point of view, HAP goes through the substitution of several ions under biological conditions which has an impact on its physicochemical properties, such as solubility. The increase or decrease in HAP solubility has shown an alteration in the biological microenvironment ion concentration, e.g., PO_4_^3−^ or Ca^2+^, improving bone formation or preventing undesirable nanoparticle accumulation in cells and tissues, but in other cases dysregulating ion homeostasis on important organs, such as those compressing the cardiovascular system [116]. Ion substitution with CO_3_^2−^ has shown a direct impact on HAP solubility/dissolution rate, the physical and chemical adsorption of biomolecules, and even the nanoparticle morphology. These observations prompt us to pay attention to the chemical structure and properties of HAP nanoparticles to exert specific functions to achieve and avoid adverse effects.

Another important feature discussed here is the relevance in the appearance of other phases in the HAP structure, such as TCP or octacalcium phosphate, that alter the biological response and the affinity with cargo. Sintering and heat treatments can cause the appearance of different CaP phases and proportions, as well as the disappearance of other bioactive molecules, such as amide or carbonate groups. For example, TCP has less hydroxyl ions than HAP, modifying the electrostatic interactions with other molecules, e.g., drugs or cell macromolecules. Thinking beyond how the content of functional groups can determine the chemical or physical adsorption of biomolecules within HAP nanoparticles, which is a process dominated by electrostatic interactions, we must consider HAP nanoparticles’ interaction capacity with other functional groups in cellular or extracellular constituents. This interaction with plasma membrane components (e.g., phospholipids, proteins, oligosaccharides, etc.) or extracellular matrix proteins modulates cell function, altering cell responses; this phenomenon can be understood as the activation or inhibition of cell signaling pathways (e.g., proliferation, division, adhesion, reactive oxygen species production, communication, differentiation, apoptosis, etc.).

Particle surface charge plays one of the major roles in the interactions of the drug delivery system and the cell. As HAP contains a high content of positive calcium ions, hydroxyl, amino, and carboxyl groups, it is important to consider the isoelectric points and the medium in which the adsorption of cargo is happening. This is mainly because although for the adsorption of biomolecules on the HAP surface the electrostatic interactions are the most relevant, it is not always true that the interaction must be between positive and negative surfaces. In the case of BSA, it was necessary for the protein and the surface to carry negative charges for the adsorption to occur. Depending on the cargo characteristics, these processes are dominated by changes of hydration, structural rearrangements, entropy, and the co-adsorption of electrolytes. Nevertheless, when we refer to HAP nanoparticles’ interaction with cell membrane, positively charged ones were more easily taken up by cells, due the higher surface charge density of the nanoparticles, resulting in a higher degree of electrostatic interactions. Depending on the specific application of the nanoparticle as a delivery system through blood circulation, for bone regeneration, to be maintained outside the cell or to be endocyted/phagocyte by somatic/immunological cells, it is important to consider which groups are meant to be presented in the nanoparticle that will interact via electrostatic forces to set the expected charges of each groups during synthesis, adsorption processes, or in vitro/in vivo applications.

For comparative studies regarding specific surface areas, particle size and morphology, some experiments carried out with BSA showed a higher protein adsorption rate with a higher specific surface area, while others showed less adsorption with the same protein [7,85]. Based on the physicochemistry of the nanoparticles, it has been established that the absorbed amount of a cargo is a function of the functional groups, porosity, surface area, pH, and the surrounding environment [18]. In rod HAP or porous HAP nanoparticles, hydrogen bonds or Van der Waals forces enhance these interactions due to a higher superficial area, allowing cell biomolecules to interact with HAP, improving some mechanisms such as cellular infiltration and adhesion (e.g., fibroblast, osteoblast). Several authors found that nanoparticles of 50 nm in size crossed the cell membrane faster than those smaller than 15 nm or bigger than 200 nm, or that biofilms containing 20 nm HAP nanoparticles reduced cell proliferation, which is a reason why different application purposes require different optimal particle sizes.

Finally, based on the physicochemical roots, it is still necessary to understand how HAP nanoparticles interact in a biological environment (cell, tissue, organ, or system), and it is important that they are not just thought of as a structure that will be accumulated, internalized, or used to deliver a cargo, but one that will directly or indirectly modulate cell signaling pathways, to improve or worsen a specific function, through the cell/HAP functional groups contained in both. With this in mind, multidisciplinary efforts to develop HAP nanoparticles as an advanced biomaterial will be a precondition to satisfy this aim. 

## Figures and Tables

**Table 1:** Vibration frequencies in FTIR spectrums from different HAP samples sintered and treated at different temperatures.

Sintering Temperature	-	-	60 °C	600 °C	950 °C	1250 °C	1350 °C
Sample	Natural HAP (cm^−1^)	Si-HAP (cm^−1^)	HAP-p (cm^−1^)	HAP (cm^−1^)	HAP (cm^−1^)	HAP (cm^−1^)	HAP (cm^−1^)
	1540	-	1540	1462.48	1456	-	1540
C-O	1548	-	-	-	-	-	-
	1418	-	-	1418.2	876	-	-
C=O	1653	-	1650	1621.94	-	-	-
C-N	1560	-	1560	-	-	-	-
N-H	1560	-	1560	-	-	-	-
Peptide	-	-	1400	-	-	-	-
P-O	1087	1089	1020	1100	1091	1100	1087
O-H	3569	3570	-	3571	3572	3575	-
SiO_4_^4−^	-	881	-	-	-	-	-
	-	498	-	-	-	-	-
Reference	[30]	[31]	[32]	[6]	[30]	[7]	[28]

HAP: hydroxyapatite, Si-HAP: SiO_4_^4−^ substituted HAP, HAP-p: HAP conjugated with peptide.

**Table 2:** Observed and assigned Raman bands from different HAP samples and natural bone.

		Synthetic HAP	Carbonated HAP	Mesoporous HAP NPs 80 °C	HAP 1400 °C
		(cm^−1^)	(cm^−1^)	(cm^−1^)	(cm^−1^)
PO_4_^3−^	^v^2	432	431	432	N/A
		447	449	445	
PO_4_^3−^	^v^4	585	581	577	N/A
		597	592	590	
		614	608	608	
		622	-	-	
A CO_3_^2−^	v_4_	-	664	-	N/A
B CO_3_^2−^	v_4_	-	730	-	
TCP					939
					946
					968
					1008
PO_4_^3−^	v_1_	962	963	959	955
		-	-	-	-
		-	-	-	-
PO_4_^3−^	v_3_	1030	-	-	1026
		1046	1048	1046	1047
		1077	1078	1072	1075
		-	-	-	1091
B CO_3_^2−^	v_1_	-	1069	1050	-
A CO_3_^2−^	v_1_	-	1114	1100	-
Reference		[47]	[50]	[51]	[30]

HAP: hydroxyapatite, TCP: tricalcium phosphate.

**Table 3:** List of peaks observed in the XRD pattern of HAP. Reproduced with permission from [10], Wiley Online Library, 2010.

2θ	d	Miller Index
(°)	(Å)	
16.84	5.250	(101)
18.78	4.720	(110)
21.60	4.070	(200)
22.97	3.880	(111)
	3.510	(201)
25.90	3.440	(002)
28.22	3.170	(102)
29.14	3.080	(210)
31.86	2.814	(211)
32.20	2.778	(112)
32.90	2.720	(300)
34.22	2.631	(202)
35.51	2.528	(301)
39.26	2.296	(212)
39.86	2.262	(310)
	2.228	(221)
42.10	2.148	(311)
	2.134	(302)
43.80	2.065	(113)
	2.040	(400)
45.32	2.000	(203)
46.69	1.943	(222)
48.16	1.890	(312)
48.59	1.871	(320)
49.51	1.841	(213)
50.53	1.806	(321)
51.33	1.780	(410)
52.24	1.754	(402), (303)
53.27	1.722	(004), (411)
54.45	1.684	(104)
55.87	1.644	(322), (223)
57.15	1.611	(313)
58.17	1.587	(501), (204)
60.01	1.542	(420)
60.45	1.530	(331)
61.66	1.503	(214), (421)
63.07	1.474	(502)
	1.465	(510)

## Data Availability

Not applicable.
